# The association between migraine and physical exercise

**DOI:** 10.1186/s10194-018-0902-y

**Published:** 2018-09-10

**Authors:** Faisal Mohammad Amin, Stavroula Aristeidou, Carlo Baraldi, Ewa K. Czapinska-Ciepiela, Daponte D. Ariadni, Davide Di Lenola, Cherilyn Fenech, Konstantinos Kampouris, Giorgos Karagiorgis, Mark Braschinsky, Mattias Linde, European Headache Federation School of Advanced Studies (EHF-SAS)

**Affiliations:** 1 Department of Neurology, Danish Headache Center, Rigshospitalet Glostrup, University of Copenhagen, Valdemar Hansens Vej 5, 2600 Glostrup, Denmark; 2 1st Neurology of Department, Eginition Hospital, School of Medicine, National and Kapodistrian University of Athens, Athens, Greece; 3 Department of Diagnostic, Medical Toxicology, Headache and Drug Abuse Research Center, Clinical and Public Health Medicine, University of Modena and Reggio Emilia, Modena, Italy; 4 Epilepsy and Migraine Treatment Centre, Kraków, Poland; 5 Department of Medico-Surgical Sciences and Biotechnologies, Sapienza University of Rome Polo Pontino, Latina, Italy; 6 Headache Centre, Guys and St Thomas NHS Trust, London, UK; 7 Neurology Clinic’s Headache Clinic, Tartu University Clinics, Tartu, Estonia; 8 Department of Neuromedicine and Movement Science, NTNU Norwegian University of Science and Technology, Trondheim, Norway; 9 Norwegian Advisory Unit on Headache, St Olavs University Hospital, Trondheim, Norway

**Keywords:** Migraine pathophysiology, Migraine treatment, Exercise headache

## Abstract

**Background:**

There is an unmet need of pharmacological and non-pharmacological treatment options for migraine patients. Exercise can be used in the treatment of several pain conditions, including. However, what exact role exercise plays in migraine prevention is unclear. Here, we review the associations between physical exercise and migraine from an epidemiological, therapeutical and pathophysiological perspective.

**Methods:**

The review was based on a primary literature search on the PubMed using the search terms “migraine and exercise”.

**Results:**

Low levels of physical exercise and high frequency of migraine has been reported in several large population-based studies. In experimental studies exercise has been reported as a trigger factor for migraine as well as migraine prophylaxis. Possible mechanisms for how exercise may trigger migraine attacks, include acute release of neuropeptides such as calcitonin gene-related peptide or alternation of hypocretin or lactate metabolism. Mechanisms for migraine prevention by exercise may include increased beta-endorphin, endocannabinoid and brain-derived neurotrophic factor levers in plasma after exercise.

**Conclusion:**

In conclusion, it seems that although exercise can trigger migraine attacks, regular exercise may have prophylactic effect on migraine frequency. This is most likely due to an altered migraine triggering threshold in persons who exercise regularly. However, the frequency and intensity of exercise that is required is still an open question, which should be addressed in future studies to delineate an evidence-based exercise program to prevent migraine in sufferers.

## Review

Migraine is the most common neurological disorder affecting around 15% of the European population [1]. It is a cyclic disorder characterized by recurrent attacks of headache accompanied by nausea, photo- and phonophobia. Usually the headache is aggravated by physical activity [2]. In some patients, attacks are accompanied by an aura with focal neurological symptoms [3]. Moreover, migraine is a disabling condition causing absenteeism from work and social life [4] and large monetary losses in society of approximately 1200 Euro per person annually [5]. Furthermore, it is a risk factor for cardiovascular diseases [6]. Although physicians possess several pharmacological options to treat migraine attacks [7], these drugs are often not effective for the individual patient, and they may have side effects. In addition, lack of adherence is a phenomenon often observed among patients who are prescribed prophylactic drugs [8]. Thus, there is an unmet need for evidence-based non-pharmacological approaches to complement pharmacotherapy in migraine prevention.

Exercise can be used for management of several chronic pain conditions [9]. Interestingly, this includes conditions comorbid with migraine such as depression, anxiety and sleep disturbances [10]. Moreover, exercise has been shown to improve self-esteem which is also associated with alleviating symptoms of migraine [10].

On the other hand, some migraineurs report exercise as a triggering factor for their attacks [10–12]. This might be a reason why the interictal behaviour of migraine patients frequently includes reduced physical activity [2, 10]. Moreover, pain aggravation induced by routine physical activity is typically reported by patients during migraine attacks [2] and is therefore included in the diagnostic criteria of migraine according defined by the International Headache Society [3].

The purpose of this review is to investigate whether recommendations for exercise in migraine are based on sufficient data and to assess what role exercise might play as a trigger for migraine and as a preventative non-pharmacologic treatment. We here define exercise as planned, structured, and repetitive bodily movements that are performed to improve or maintain physical fitness [9]. This review is intended to help establish a proper exercise strategy for patients with migraine enabling clinicians to improve their migraine management strategies.

## Methods

An initial literature search was performed up to November 15th, 2017 on PubMed.com. using “migraine and exercise” as search terms and applying the “humans” filter. It resulted in 280 hits. All titles were screened, and we excluded papers where the content was not relevant to the topic. Secondly, the remaining abstracts (*N* = 150) were assessed by the first author. Again, all abstract without relevant content, for the topic, were excluded. The remaining articles (*N* = 44) were divided, according to the content, between authors of each section in the review. Every author added additional papers when needed in their respective section.

### Associations between physical exercise and migraine—epidemiological evidence

Various large population-based studies have concluded that low physical activity levels are associated with higher prevalence and frequency of migraine and other headaches [13–15]. Vice versa, higher physical activity levels are associated with reductions in migraine headache frequency and and with less migraine-related disability [16–19].

The study by Varkey et al. [14], using individuals from one of the world’s largest epidemiological studies, the Nord-Trøndelag Health Survey (HUNT), was divided in two parts, using a prospective and a cross sectional design. In the first part of the study (1984–1986) a total of 22,397 participants, characterized as headache-free and analgesic drug-free, answered a questionnaire on exercise. Eleven years later the participants answered a questionnaire on physical activity and headache. Physically active individuals reported less non-migraine headaches than physically inactive individuals. A total of 46,648 participants were included in the cross-sectional part of the study. Migraine as well as non-migraine headache was more prevalent in groups reporting low physical activity [14].

Molarius et al. [20] conducted a Swedish population study comprising a random sample of 43,770 men and women, aged 18–79 years, covering 5a vast area of 58 municipalities throughout the country. The aim of the study was to evaluate the relationship between socio-economic factors, lifestyle habits, and recurrent headaches/migraine reported by the patients. Results showed that physically inactive subjects had a higher prevalence of self-reported migraine and/or recurrent headache than physically active subjects [20].

Hagen et al. [21], found a lower mean peak oxygen uptake (VO_2_-peak) among patients with migraine and tension-type headache than those who were headache-free. An increase of VO_2_-peak but not migraine frequency was reported after a 12-week intervention with regular exercise [21].

Kinart et al. [22], assessed 791 American first division male and female basketball players, and demonstrated a lower prevalence of migraine compared to the general population. Whether playing basketball prevented migraines or predisposition to migraine prevented the individual from becoming an elite basketball player can be discussed [22].

In a Korean study by Seok et al. [23], 136 patients with *transformed migraine* (TM) were followed for at least 1 year. Authors defined TM as a migraine attack frequency greater than 15 per month over a period of at least 6 months. The migraine diagnosis met the ICHD-II diagnostic criteria [24]. This study also included patients with medication overuse defined as the intake of simple analgesics for *&gt;* 15 days/month for 3 months, or a combination of analgesics, ergotamine, or triptan on *&gt;* 10 days/month for 3 months. The aim of the study was to identify the factors (i.e discontinuation of overused drugs, regular intake of preventive drugs and lifestyle modification, including regular exercise and no use of caffeine, alcohol and tobacco) that play a role in the reversal of TM to episodic migraine (defined by authors as less than 8 headache days per month). A total of 95 patients (70%) converted from TM to episodic migraine. Factors that significantly differed between those who converted to episodic migraine and those who did not were regular intake of preventative drugs (*p* &lt; 0.001), discontinuation of overused drugs (*p* &lt; 0.001), and regular exercise (*p* = 0.04) were. However, whether regular exercise caused improvement of migraine status and subsequently caused reduction of medication overuse or effect of preventative drugs and discontinuation of medication overuse caused less migraine and thus more exercise, has not been discussed in the study.

### Exercise as a trigger factor for migraine attacks—clinical evidence

The number of studies demonstrating exercise as a trigger factor for migraine is relatively limited. Williams et al. [25] explored the prevalence of exercise-triggered migraine in a cohort of 129 New Zealanders University students aged between 15 and 40 years. Eleven (9%) of them had suffered a headache fulfilling the ICHD-I criteria [26] for migraine during or shortly after physical activity [25]. Later, in a larger prospective clinic-based study from the USA on 1207 patients aged between 13 and 80 years (mean age, 37.7 ± 12.0 years), exercise was reported to be a trigger factor for migraine among 22% [27]. However, recent smaller studies, examining more specifically exercise as a trigger factor for migraine reported even higher proportions [28–30]. A Dutch prospective study conducted on 103 persons with migraine, reported a lifetime prevalence of exercise-triggered migraine attacks at 38% among migraine patients. The pain started during exercise in 17 patients out of 30 (56%) and caused exercise discontinuation [28]. Additionally, a Danish study examining experimental provocation of migraine attacks using self-reported natural trigger factors, showed that migraine could be triggered by an intense run or by exercise on an ergometer bike in 4 patients out of 12, who reported exercise as their triggering factor [30]. In a recent Swedish rest-retest study on patients attending a tertiary headache centre and reporting exercise as migraine attacks trigger. Eight (57%) out of 14 patients who completed test-rested reported a migraine attack after intensive aerobic exercise (indoor cycling) after the first test and 3 (21%) after both tests [29]. Moreover, the baseline attack frequency was higher in the 8 patients who developed attack after the first test compared to the 6 patients who were not able to trigger attack by exercising (*p* = 0.036) [29].

Some studies pointed out the high comorbidity of migraine with primary exertional headache (PEH), which is a relatively uncommon primary headache disorder which occurs particularly in hot weather or at high altitude, lasting within 48 h brought on exclusively during or after strenuous physical exertion, with a divergent prevalence of between 0.2–12.3% among the general population [31]. PEH and migraine comorbidity might be relatively common in middle-aged migraine patients: Hanashiro et al. [32] found a co-existence of PEH and migraine in 158 out of 2546 patients (6.2%). Indeed, PEH prevalence is even higher according with other studies: Chen et al. [33] stated a prevalence of the 30% among adolescent migraineurs, similarly to Ende-Kastelijn et al. [31], who found a prevalence of 26% [33]. These authors concluded that, despite the classification, PEH and exercise-triggered migraine attacks are quite similar, with PEH attacks that could be migraine attacks per se [31, 33]. In a study investigating exertional headache prevalence and characteristics in 1963 Taiwanese adolescents, Chen et al. [33] reported that exertional headache was seen more frequently in persons with migraine compared to those without migraine [54.9% vs. 25.7%, odds ratio (OR) 3.4*, p* &lt; 0.001]. Moreover, exercise-triggered headache accompanied by nausea or vomiting in 136 migraineurs (41.6%) and in only 52 none-migraineurs (19.3%) (*p* &lt; 0.001), whilst photophobia and phonophobia were reported by 36 migraineurs (11.0%) and only by 7 none-migraineurs (2.6%) *(p* &lt; 0.001). It can be discussed whether the headache provoked by exercise in migraine patients was exertional headache or exercise-induced migraine attacks.

### Exercise as acute treatment of migraine

To the best of our knowledge nearly all studies have explored the efficacy of exercise as prophylactic treatment. The data identified on exercise as acute treatment of migraine were in the form of case reports such as that of a 43-year-old Caucasian woman with episodic migraine with aura who aborted her attacks by running during the prodromal phase [34]. Another case story [35] reported successfully abortion of the attack by fast intensive running. The author suggested that exercise effectiveness could be due to a macro-mechanism on the blood vessels and a micro-mechanism settling the hormone imbalance leading to migraine attack. Clinical trials are needed to confirm the hypotheses from the clinical observations, that exercise, in some, can be used as acute treatment.

### Exercise as prophylactic treatment of migraine

Exercise plays an imperative role in the management of several chronic diseases as it prevents or reduces different kinds of chronic pain (chronic neck or low back pain, osteoarthritis, headache, fibromyalgia) [9]. The efficacy of exercise as prophylactic treatment for migraine has been investigated in several studies. Some of the studies report a significant reduction in pain intensity as well as beneficial effects on frequency and duration of migraine attacks, without reporting worsening of migraine [16, 17, 36–40]. In a cross-sectional, community-based study of 480 medical students, Domingues et al. [19] revealed a significantly lower migraine associated disability, assessed by the MIDAS scores (*p* = 0.03) between those who practiced regularly exercise (MIDAS: 15.49 ± 1.78) to those who did no exercise (MIDAS: 8.81 ± 1.40). Physical exercise included both aerobic and strength training. However, no difference in migraine prevalence between the two groups was found [19]. A German single-arm longitudinal study investigated the effect of a 10-week aerobic endurance programme on headache and cognitive function [39]. Significant reduction in the numbers of migraine days per month (*p* = 0.01) and migraine attacks per month (*p* = 0.001) was reported. Moreover, the cognitive function (i.e. information processing and attention) was also improved between the assessment 8 weeks before and after the exercise therapy period. Varkey et al. [17] developed an aerobic exercise programme in Sweden for untrained patients suffering from migraine, and showed that it could safely improve exercise capacity (increasing maximum oxygen uptake) without making their migraines worse [17]. Twenty-six patients followed a 12-week program based on indoor cycling, including warm-up and cool-down periods. The program was well tolerated with no deterioration of migraine status and significant improvements in attack frequency, intensity, quality of life and intake of medicine [17]. This was followed by a 3-arm randomized, controlled trial in 91 migraine patients (migraine frequency 2–8 days/month) comparing submaximal aerobic exercise three times a week for 3 months to topiramate or relaxation. All three interventions proved beneficial and equivalent with respect to the frequency of migraine attacks, but adverse events appeared only in the topiramate group [18].

In a later randomized comparative study in Brazil, it was concluded that the combination of amitriptyline and aerobic exercise, resulted in a greater reduction in frequency, duration and intensity of headache, and depression and anxiety scores compared to amitriptyline alone among patients with chronic migraine [40]. Krøll et al. [41] recently conducted a randomized, controlled, clinical trial in Denmark evaluating the effect of a three-month aerobic exercise involving cross-training, biking and brisk walking on 26 persons with migraine and co-existing tension-type headache and neck pain [41]. Exercise caused a reduction burden of migraine and improved ability to engage in physical activity. Migraine frequency, pain intensity and duration, were also reduced, but not significantly compared to controls.

There is limited evidence concerning the role of non-aerobic types of exercise in the treatment of migraine. In a randomized controlled trial 72 migraine without aura patients were randomly assigned yoga therapy or self-care. A significant reduction in migraine frequency was reported in the yoga versus self-care group (*p* &lt; 0.001) [42]. Moreover, a Japanese single-arm pilot study of 6 migraine patients, reported a 50% reduction of migraine frequency in 5 out of 6 subjects [43].

### Pathophysiological explanations/theories of mechanism for the useful and problematic associations between physical exercise and migraine

This chapter discusses possible pathophysiological theories underlying I. exercise as a trigger factor for migraine, II. aggravation of acute migraine pain by physical activity, and III. the previously described therapeutic effects of exercise in migraine.

#### I. Mechanisms for triggering of migraine attacks by exercise

##### Dysfunction of the neuropeptide hypocretin

Hypocretin is produced by the hypothalamus and is involved in regulation of sleep and arousal [28]. Several functions of hypocretins are impaired in patients with migraine and may be involved in the pathophysiology of the premonitory symptoms preceding a migraine attack such as excessive sleepiness, food cravings, yawning and fatigue [44]. Patients often report that sleep can abort a migraine attack [45]. Moreover, altered and disturbed sleep is reported in triathletes who have overreached [46]. Thus, vigorous exercise could through influence of the hypocretin pathway theoretically initiate attacks [28].

##### Unfavourable energy metabolism due to lactate

Anaerobic exercise results in the byproduct lactate. Magnetic resonance spectroscopy has shown higher migraine frequency is related to increased brain lactate levels [47].

##### Calcitonin gene-related peptide (CGRP)

CGRP is a neuropeptide found extensively in the central and peripheral nervous system that results in vasodilation and sensory transmission of pain pathways [48]. It is increased in pain conditions [49] and has been shown to be released during migraine attacks and return to normal levels after the use of the migraine abortive medication sumatriptan [50]. During exercise, CGRP levels rise, and it is believed that it may be associated with the increased pain experience in delayed onset muscle soreness [51]. However, CGRP has not been measured during exercise in people with migraine, so its potential role as a mediator through which strenuous exercise could trigger migraine attacks needs further corroboration.

#### II. Mechanisms for aggravation of acute migraine pain

The perivascular nerve afferents from the trigeminal nerve are activated during a migraine attack [52], leading to release of pro-inflammatory substances that may sensitize the tissue surrounding arteries particularly in the meninges [53]. Thus, normal pulsations, which are even not sensed under normal conditions, may be experienced as pain during the migraine attack. In this setting, every activity that increases heart rate and/or arterial flow causes increased pulsations experienced as the throbbing pain by the patients. Most people with migraine, in contrast to those with tension-type headache, therefore avoid routine physical activity during migraine pain [2]. On the contrary, lack of temporal relationship between ictal throbbing and arterial pulse [54], suggests a minor role of meningeal arterial pulsation in pain aggravation. Another possible underlying mechanism could be increased intracranial pressure during migraine attacks [55]. Coughing can increase the intracranial pressure dramatically. However, coughing did not aggravate the pain as much as bending forward in one study [2].

#### III. Mechanisms for therapeutic effects of exercise in migraine

Endogenous opioids modulate pain and were found to be lower during migraine attack than in the pain free period and their concentration arise at the end of the attack [56]. After exercise beta-endorphin significantly increase only when anaerobic threshold was exceeded [57–60] or if an exercise, at a lower threshold, was prolonged for about 50 min [61]. Beta-endorphin, is an endogenous opioid, which is produced by the anterior pituitary and results in analgesia by binding to pre- and postsynaptic opioid receptors (mainly mu receptors) [62]. In the peripheral nervous system, it seems to inhibit the release of substance P thus decreasing the transmission of pain pathways whereas in the central nervous system it acts presynaptic to inhibit the release of GABA [63, 64]. This results in excess production of dopamine which is associated with pleasure [65]. Beta-endorphin levels have been found to be lower in patients with migraine in comparison to healthy controls [66]. This opioid is even lower in patients with chronic migraine [67]. However, exercise results in increased beta-endorphin levels [68]. Köseoglu et al. [16] studied 40 female migraine without aura patients, who exercised for 6 weeks, 40–50 min 3 times per week at 60–80% of their maximal heart rate during headache free periods. Beta-endorphin xlevels were drawn before and after the exercise program and doubled from pre- to post-exercise levels. Exercise resulted in an increase in the beta-endorphin which possibly lead to less headache days – decreased from two to one headache days per month [16].

The endocannabinoid ligand anandamide (AEA), a precursor of the endocannabinoid system, increases following exercise and is thought to result in a “runners high” [69]. AEA levels are increased in high-intensity endurance running but not in low-intensity walking [70]. The “runners high” is a sudden positive feeling of euphoria, sedation, analgesia and anxiolysis. Levels of AEA rise and result in the release of cannabinoids 1 (CB1) and 2 (CB2). In rats, Fuss et al. [71] showed that anxiolysis was mediated through the CB1 receptors, whereas CB1 and CB2 receptors mediated pain reduction. In migraine patients, this endocannabinoid reward system is dysfunctional and concentrations of AEA are significantly lower than normal controls possibly contributing to sensitization of the trigeminal and spinal pathways [72, 73]. Exercise can have an important role in the modulation of pain processing from an affective-motivational perspective though the activation of endogenous cannabinoid signaling [69, 74–76]. No studies have looked at variations in AEA in migraine patients who exercise.

Brain-derived neurotrophic factor (BDNF) is a polypeptide, related to polypeptide growth factors which are thought to be involved in growth, differentiation and survival of neurons [77–80]. Release of BDNF from the trigeminal ganglion neurons is induced by inflammatory mediators, such as CGRP, and results in altered plasticity of neural pathways [81]. Serum levels of BDNF have been shown to be statistically higher during migraine attacks than in interictal period [82, 83]. In migraine, BDNF may be upregulated and may play a role in sustained mechanisms of central sensitization of pain pathways [84]. In humans, BDNF levels increase after exercise [85–87]. High BDNF levels following exercise are thought to prevent neuronal loss and have positive effects on cognitive function in animal studies [88]. No studies have looked at variations in BDNF in migraine patients who exercise.

Beta blockers, and angiotensin-2 inhibitors can be used as a prophylaxis for migraine. Multiple modes of actions have been postulated including reduction of neuronal firing of noradrenergic neurons from the locus coeruleus [89], regulation of the firing rate of GABA from the periaqueductal grey matter [90] and blockage of some serotonin receptors [89]. However, in maintaining a regular blood pressure by decreasing cardiac output and decreasing blood pressure these prophylactics may prevent migraine activity as migraine patients are known to have impaired autonomic control of cerebral vasoreactivity [91]. Altered blood pressure and cardiac output may therefore be key mechanisms through which exercise have a prophylactic effect on migraine.

Nitric oxide (NO) is a potent vasodilator which is known to regulate cerebral blood flow [92]. Glyceryl trinitrate, its prodrug, is known to produce headaches in healthy volunteers. In migraine, glyceryl trinitrate is thought to act via liberation of NO within the neurovascular system [93]. Fitness is known to regulate vascular tone [34], and is linked to a rise is NO level. This increase may also protect the endothelium by reducing norepinephrine [94], and preventing the production of vasoconstrictors and free radicals in vessel walls [95]. In a Turkish study, 40 females with migraine without aura were assigned to an active group (i.e. 1 h of moderate submaxminal aerobic exercise three times per week) or a control group (i.e. medication only). In the active group headache frequency decreased from 7.4 (standard deviation [SD]) 2.9) to 3.6 (SD 1.6) days (*p* &lt; 0.05) whereas in the control group it changed from 8.9 (SD 3.3) to 7.0 (SD 2.4) days (*p* &lt; 0.05). Pain score also decreased from 8.8 (SD 1.7) to 4.0 (SD 1.4) on the visual analogue scale from 0 to 10 (0 = no pain and 10 = worst imaginable pain) in the exercise group (non-significant) versus 8.5 (SD 0.8) to 7.0 (SD 0.9) in the control group (non-significant). However, the pain intensity was significantly more reduced in the exercise versus the control group (*p* &lt; 0.05). Blood NO was measured before and after the exercise programme. The NO level in the active group changed from a baseline of 13.52 (SD 3.62) to 19.63 (SD 5.30) after the 8-week programme. The NO level in the control group was 16.20 (SD 6.03) at baseline and 13.16 (SD 6.00) after 8 weeks. There was no significant difference between the groups (*p* &gt; 0.05) (37).

Repeated aerobic exercise has been shown to be beneficial in sleep regulation, weight management, mood and cardiovascular function [74, 96]. This model postulates that if one engages in aerobic exercise, migraine burden is altered by decreasing pro-inflammatory markers and increasing anti-inflammatory markers in the brain. Also from a psychological and behavioural point of view one might develop increased self-efficacy and increased outcome expectations from exercise. Thus, people who adhere to exercise despite barriers may become more capable, confident and competent at managing their migraine [97, 98]. However, the underlying biological mechanisms for any such processes are unknown.

### Implications for populational health—conclusions and recommendations

To sum up, the high prevalence of migraine [99], as well as the important associated socioeconomic burden for patients and societies in general [100, 101], emphasizes the unmet need of novel therapeutic options to improve the efficacy and populational coverage of migraine prophylaxis.

Increasing numbers of comorbidities, such as depression, anxiety [102, 103] and obesity [104] have been associated with migraine. Thus, non-pharmacological treatments become even more evident to avoid polypharmacy or drug interactions. Moreover, there are also patients in whom migraine attacks are refractory to pharmacological treatment [105]. Regular exercise has been proposed as a possible therapeutic option for migraine. Advantages are that it is available to most people with migraine, also in low-and-middle-income countries, with low physician coverage, that it costs nothing or very little, and that it has general health benefits and should be performed by everyone.

Scientific research in this field, although limited, indicates positive results, showing that aerobic exercise training can have positive therapeutic outcomes for adolescent and adult migraine patients, reducing the frequency and the intensity of the headaches, body weight and psychiatric comorbidities, as well as improving the quality of life of these patients [17, 38, 106] and conferring multiple health benefits (weight, sleep regulation, mood, cardiovascular function), including conditions that are frequently comorbid with migraine (obesity, hypertension, sleep apnoea, depression, anxiety) [18], without causing side effects and without significant costs [107]. Moreover, an exercise intervention may prove suitable for people with migraine considering their tendency toward inactivity [108] and the direct association between low physical activity and greater migraine frequency [14]. However more research is needed, especially controlled studies with long-term follow up, for the generalization of these results.

The empirical support for recommending a specific exercise program for prophylactic treatment is relatively limited but we can provide a general guide. Aerobic exercises like cycling and walking, are preferred over eccentric or isometric muscle work and a warm-up period should be included considering that both high intensity exercise and insufficient warm-up are reported to be common triggering factors for migraine [9]. The program in total should remain in a tolerable level preventing exercise-related pain and disability, with a suggested frequency of two to three times per week. Patients must continue with this despite initial lack of improvement because it is found that people with migraine can develop a “tolerance” to the pain-inducing effects of moderate exercise [109].

## Conclusions

In conclusion, considering this combination of efficacy, minimal side effects, multiple health benefits and cost savings, exercise programs seem to be an important asset in the management of migraine and it is encouraged that public health services financially support such interdisciplinary intervention programs and educational campaigns and that headache experts, as well as general practitioners, incorporate them within the therapeutic plan for their patients.

## Abbreviations

AEA: Endocannabinoid ligand anandamide
BDNF: Brain-derived neurotrophic factor
CB1: Cannabinoid receptor type 1
CB2: Cannabinoid receptor type 2
CGRP: Calcitonin gene-related peptide
GABA: Gamma-aminobutyruc acid
HUNT: The Nord-Trøndelag Health Survey
ICHD: International Classification of Headache Disorders
MIDAS: Migraine disability assessment test
NO: Nitric oxide
OR: Odds ratio
PEH: Primary exertional headache
SD: Standard deviation
TM: Transformed migraine
VO_2_-peak: The maximum rate of oxygen consumption measured during incremental exercise

## References

[1] Stovner LJ, Andree C (2010). Prevalence of headache in Europe: a review for the Eurolight project. J Headache Pain.

[2] Martins IP, Gouveia RG, Parreira E (2006). Kinesiophobia in migraine. J Pain.

[3] Headache Classification Committee of the International Headache Society (2013). The international classification of headache disorders, 3rd edition (beta version). Cephalalgia.

[4] Leonardi M, Raggi A, Ajovalasit D, Bussone G, D’Amico D (2010). Functioning and disability in migraine. Disabil Rehabil.

[5] Linde M, Gustavsson A, Stovner LJ, Steiner TJ, Barré J, Katsarava Z, Laínez JM, Lantéri-Minet M, Rastenyte D, Ruiz de la Torre E, Andrée C, Steiner TJ (2012). The cost of headache disorders in Europe: the Eurolight project. Eur J Neurol.

[6] Sacco S, Ricci S, Carolei A (2012). Migraine and vascular diseases: a review of the evidence and potential implications for management. Cephalalgia.

[7] Evers S, Áfra J, Frese A, Goadsby PJ, Linde M, May A, Sándor PS, European Federation of Neurological Societies (2009). EFNS guideline on the drug treatment of migraine--revised report of an EFNS task force. Eur J Neurol.

[8] Linde M, Jonsson P, Hedenrud T (2008). Influence of disease features on adherence to prophylactic migraine medication. Acta Neurol Scand.

[9] Daenen L, Varkey E, Kellmann M, Nijs J (2015). Exercise, not to exercise, or how to exercise in patients with chronic pain? Applying science to practice. Clin J Pain.

[10] Irby MB, Bond DS, Lipton RB, Nicklas B, Houle TT, Penzien DB (2016). Aerobic exercise for reducing migraine burden: mechanisms, markers, and models of change processes. Headache.

[11] Nadelson C (2006). Sport and exercise-induced migraines. Curr Sports Med Rep.

[12] Lane JC (2000). Migraine in the athlete. Semin Neurol.

[13] Queiroz LP, Peres MFP, Piovesan EJ, Kowacs F, Ciciarelli MC, Souza JA, Zukerman E (2009). A nationwide population-based study of migraine in Brazil. Cephalalgia.

[14] Varkey E, Hagen K, Zwart JA, Linde M (2008). Physical activity and headache: results from the Nord-Trøndelag health study (HUNT). Cephalalgia.

[15] Wöber C, Brannath W, Schmidt K, Kapitan M, Rudel E, Wessely P, Wöber-bingöl C, PAMINA Study Group (2007). Prospective analysis of factors related to migraine attacks: the PAMINA study. Cephalalgia.

[16] Köseoglu E, Akboyraz A, Soyuer A, Ersoy AÖ (2003). Aerobic exercise and plasma beta endorphin levels in patients with migrainous headache without aura. Cephalalgia.

[17] Varkey E, Cider Å, Carlsson J, Linde M (2009). A study to evaluate the feasibility of an aerobic exercise program in patients with migraine. Headache.

[18] Varkey E, Cider Å, Carlsson J, Linde M (2011). Exercise as migraine prophylaxis: a randomized study using relaxation and topiramate as controls. Cephalalgia.

[19] Domingues RB, Teixeira AL, Domingues SA (2011). Physical practice is associated with less functional disability in medical students with migraine. Arq Neuropsiquiatr.

[20] Molarius A, Tegelberg A, Ohrvik J (2008). Socio-economic factors, lifestyle, and headache disorders - a population-based study in Sweden. Headache.

[21] Hagen K, Wisløff U, Ellingsen Ø, Stovner LJ, Linde M (2015). Headache and peak oxygen uptake: the HUNT3 study. Cephalalgia.

[22] Kinart CM, Cuppett MM, Berg K (2002). Prevalence of migraines in NCAA division I male and female basketball players. National Collegiate Athletic Assoc Headache.

[23] Jung IS, Hyung IC, Chung CS (2006). From transformed migraine to episodic migraine: reversion factors. Headache.

[24] Headache Classification Committee of the International Headache Society (2004). The international classification of headache disorders. Cephalalgia.

[25] Williams SJ, Nukada H (1994). Sport and exercise headache: part 2. Diagnosis and classification. Br J Sports Med.

[26] Sjaastad O (1988). Classification and diagnostic criteria for headache disorders, cranial neuralgias and facial pain. Headache classification Committee of the International Headache Society. Cephalalgia.

[27] Kelman L (2007). The triggers or precipitants of the acute migraine attack. Cephalalgia.

[28] Koppen H, van Veldhoven PLJ (2013). Migraineurs with exercise-triggered attacks have a distinct migraine. J Headache Pain.

[29] Varkey E, Grüner Sveälv B, Edin F, Ravn-Fischer A, Cider Å (2017). Provocation of migraine after maximal exercise: a test-retest study. Eur Neurol.

[30] Hougaard A, Amin FM, Hauge AW, Ashina M, Olesen J (2013). Provocation of migraine with aura using natural trigger factors. Neurology.

[31] Van Der Ende-Kastelijn K, Oerlemans W, Goedegebuure S (2012). An online survey of exercise-related headaches among cyclists. Headache.

[32] Hanashiro S, Takazawa T, Kawase Y, Ikeda K (2015). Prevalence and clinical hallmarks of primary exercise headache in middle-aged Japanese on health check-up. Intern Med.

[33] Chen SP, Fuh JL, Lu SR, Wang SJ (2009). Exertional headache - a survey of 1963 adolescents. Cephalalgia.

[34] Darling M (1991). The use of exercise as a method of aborting migraine. Headache.

[35] Strelniker YM (2009). Intensive running completely removes a migraine attack. Med Hypotheses.

[36] Lemstra M, Stewart B, Olszynski WP (2002). Effectiveness of multidisciplinary intervention in the treatment of migraine: a randomized clinical trial. Headache.

[37] Osün Narin S, Pinar L, Erbas D, Oztürk V, Idiman F (2003). The effects of exercise and exercise-related changes in blood nitric oxide level on migraine headache. Clin Rehabil.

[38] Dittrich SM, Günther V, Franz G, Burtscher M, Holzner B, Kopp M (2008). Aerobic exercise with relaxation: influence on pain and psychological well-being in female migraine patients. Clin J Sport Med.

[39] Overath CH, Darabaneanu S, Evers MC, Gerber WD, Graf M, Keller A, Niederberger U, Schäl H, Siniatchkin M, Weisser B (2014). Does an aerobic endurance programme have an influence on information processing in migraineurs?. J Headache Pain.

[40] Santiago MDS, Carvalho D de S, Gabbai AA, Pinto MMP, Moutran ARC, Villa TR (2014) Amitriptyline and aerobic exercise or amitriptyline alone in the treatment of chronic migraine: a randomized comparative study. Arq Neuropsiquiatr 72:851–855 10.1590/0004-282x20140148 25410451

[41] Krøll LS, Hammarlund CS, Linde M, Gard G, Jensen RH (2018) The effects of aerobic exercise for persons with migraine and co-existing tension-type headache and neck pain. A randomized, controlled, clinical trial. Cephalalgia [Epub ahead of print]. 10.1177/0333102417752119 10.1177/0333102417752119 29333870

[42] John PJ, Sharma N, Sharma CM, Kankane A (2007). Effectiveness of yoga therapy in the treatment of migraine without aura: a randomized controlled trial. Headache.

[43] Elinoff V, Lynn SJ, Ochiai H, Hallquist M (2009). The efficacy of Kiko exercises on the prevention of migraine headaches: a pilot study. Am J Chin Med.

[44] Rainero I, Rubino E, Gallone S, Fenoglio P, Picci LR, Giobbe L, Ostacoli L, Pinessi (2011). Evidence for an association between migraine and the hypocretin receptor 1 gene. J Headache Pain.

[45] Bigal ME, Hargreaves RJ (2013). Why does sleep stop migraine?. Curr Pain Headache Rep.

[46] Hausswirth C, Louis J, Aubry A, Bonnet G, Duffield R, Le Meur Y (2014). Evidence of disturbed sleep and increased illness in overreached endurance athletes. Med Sci Sports Exerc.

[47] Watanabe H, Kuwabara T, Ohkubo M, Tsuji S, Yuasa T (1996). Elevation of cerebral lactate detected by localized 1H-magnetic resonance spectroscopy in migraine during the interictal period. Neurology.

[48] Arulmani U, MaassenVanDenBrink A, Villalón CM, Saxena PR (2004). Calcitonin gene-related peptide and its role in migraine pathophysiology. Eur J Pharmacol.

[49] Onuoha GN, Alpar EK (1999). Calcitonin gene-related peptide and other neuropeptides in the plasma of patients with soft tissue injury. Life Sci.

[50] Goadsby PJ, Edvinsson L (1993). The trigeminovascular system and migraine: studies characterizing cerebrovascular and neuropeptide changes seen in humans and cats. Ann Neurol.

[51] Jonhagen S, Ackermann P, Saartok T, Renstrom PA (2006). Calcitonin gene related peptide and neuropeptide Y in skeletal muscle after eccentric exercise: a microdialysis study. Br J Sports Med.

[52] Bernstein C, Burstein R (2012). Sensitization of the trigeminovascular pathway: perspective and implications to migraine pathophysiology. J Clin Neurol.

[53] Silberstein SD (2004). Migraine pathophysiology and its clinical implications. Cephalalgia.

[54] Ahn AH (2010). On the temporal relationship between throbbing migraine pain and arterial pulse. Headache.

[55] Blau JN, Dexter SL (1981). The site of pain origin during migraine attacks. Cephalalgia.

[56] Anselmi B, Baldi E, Casacci F, Salmon S (1980). Endogenous opioids in cerebrospinal fluid and blood in idiopathic headache sufferers. Headache.

[57] McMurray RG, Forsythe WA, Mar MH, Hardy CJ (1987). Exercise intensity-related responses of beta-endorphin and catecholamines. Med Sci Sports Exerc.

[58] Goldfarb AH, Hatfield BD, Armstrong D, Potts J (1990). Plasma beta-endorphin concentration: response to intensity and duration of exercise. Med Sci Sports Exerc.

[59] Langenfeld ME, Hart LS, Kao PC (1987). Plasma beta-endorphin responses to one-hour bicycling and running at 60% VO2max. Med Sci Sports Exerc.

[60] Rahkila P, Hakala E, Alén M, Salminen K, Laatikainen T (1988). β-Endorphin and corticotropin release is dependent on a threshold intensity of running exercise in male endurance athletes. Life Sci.

[61] Schwarz L, Kindermann W (1989). β-Endorphin, catecholamines, and cortisol during exhaustive endurance exercise. Int J Sports Med.

[62] Guillemin R, Vargo T, Rossier J, Minick S, Ling N, Rivier C, Vale W, Bloom F (1977). Beta-endorphin and adrenocorticotropin are selected concomitantly by the pituitary gland. Science.

[63] Stein C (1995). The control of pain in peripheral tissue by opioids. N Engl J Med.

[64] Brunton L (2006). Goodman &amp; Gilman’ s The Pharmacological Basis of Therapeutics.

[65] Leknes S, Tracey I (2008). A common neurobiology for pain and pleasure. Nat Rev Neurosci.

[66] Sicuteri F (1978). Endorphins, opiate receptors and migraine headache. Headache.

[67] Misra UK, Kalita J, Tripathi GM, Bhoi SK (2013). Is β endorphin related to migraine headache and its relief?. Cephalalgia.

[68] Schwarz L, Kindermann W (1990). Beta-endorphin, adrenocorticotropic hormone, cortisol and catecholamines during aerobic and anaerobic exercise. Eur J Appl Physiol Occup Physiol.

[69] Sparling PB, Giuffrida A, Piomelli D, Rosskopf L, Dietrich A (2003). Exercise activates the endocannabinoid system. Neuroreport.

[70] Raichlen DA, Foster AD, Gerdeman GL, Seillier A, Giuffrida A (2012). Wired to run: exercise-induced endocannabinoid signaling in humans and cursorial mammals with implications for the “runner”s high. J Exp Biol.

[71] Fuss J, Steinle J, Bindila L, Auer MK, Kirchherr H, Lutz B, Gass P (2015). A runner’s high depends on cannabinoid receptors in mice. Proc Natl Acad Sci.

[72] Sarchielli P, Pini LA, Coppola F, Rossi C, Baldi A, Mancini ML, Calabresi P (2007). Endocannabinoids in chronic migraine: CSF findings suggest a system failure. Neuropsychopharmacology.

[73] Perrotta A, Arce-Leal N, Tassorelli C, Gasperi V, Sances G, Blandini F, Serrao M, Bolla M, Pierelli F, Nappi G, Maccarrone M, Sandrini G (2012). Acute reduction of anandamide-hydrolase (FAAH) activity is coupled with a reduction of nociceptive pathways facilitation in medication-overuse headache subjects after withdrawal treatment. Headache.

[74] Buse DC, Manack A, Serrano D, Turkel C, Lipton RB (2010). Sociodemographic and comorbidity profiles of chronic migraine and episodic migraine sufferers. J Neurol Neurosurg Psychiatry.

[75] Boecker H, Sprenger T, Spilker ME, Henriksen G, Koppenhoefer M, Wagner KJ, Valet M, Berthele A, Tolle TR (2008). The runner’s high: Opioidergic mechanisms in the human brain. Cereb Cortex.

[76] Dietrich A, McDaniel WF (2004). Endocannabinoids and exercise. Br J Sports Med.

[77] Acheson A, Conover JC, Fandl JP, Dechiara TM, Russell M, Thadani A, Squinto SP, Yancopoulos GD, Lindsay RM (1995). A BDNF autocrine loop in adult sensory neurons prevents cell death. Nature.

[78] Greenberg ME, Xu B, Lu B, Hempstead BL (2009). New insights in the biology of BDNF synthesis and release: implications in CNS function. J Neurosci.

[79] Huang EJ, Reichardt LF (2001). Neurotrophins: roles in neuronal development and function. Annu Rev Neurosci.

[80] Numakawa T, Suzuki S, Kumamaru E, Adachi N, Richards M, Kunugi H (2010). BDNF function and intracellular signaling in neurons. Histol Histopathol.

[81] Bałkowiec-Iskra E, Vermehren-Schmaedick A, Balkowiec A (2011). Tumor necrosis factor-α increases brain-derived neurotrophic factor expression in trigeminal ganglion neurons in an activity-dependent manner. Neuroscience.

[82] Tanure MTA, Gomez RS, Hurtado RCL, Teixeira AL, Domingues RB (2010). Increased serum levels of brain-derived neurotropic factor during migraine attacks: a pilot study. J Headache Pain.

[83] Fischer M, Wille G, Klien S, Shanib H, Holle D, Gaul C, Broessner G (2012). Brain-derived neurotrophic factor in primary headaches. J Headache Pain.

[84] Sarchielli P, Mancini ML, Floridi A, Coppola F, Rossi C, Nardi K, Acciarresi M, Pini LA, Calabresi P (2007). Increased levels of neurotrophins are not specific for chronic migraine: evidence from primary fibromyalgia syndrome. J Pain.

[85] Coelho FM, Gobbi S, Andreatto CA, Corazza DI, Pedroso RV, Santos-Galduróz RF (2013). Physical exercise modulates peripheral levels of brain-derived neurotrophic factor (BDNF): a systematic review of experimental studies in the elderly. Arch Gerontol Geriatr.

[86] Huang T, Larsen KT, Ried-Larsen M, Møller NC, Andersen LB (2014). The effects of physical activity and exercise on brain-derived neurotrophic factor in healthy humans: a review. Scand J Med Sci Sports.

[87] Dinoff A, Herrmann N, Swardfager W, Liu CS, Sherman C, Chan S, Lanctôt KL (2016). The effect of exercise training on resting concentrations of peripheral brain-derived neurotrophic factor (BDNF): a meta-analysis. PLoS One.

[88] Vaynman S, Ying Z, Gomez-Pinilla F (2004). Hippocampal BDNF mediates the efficacy of exercise on synaptic plasticity and cognition. Eur J Neurosci.

[89] Galletti F, Cupini LM, Corbelli I, Calabresi P, Sarchielli P (2009). Pathophysiological basis of migraine prophylaxis. Prog Neurobiol.

[90] Xiao C, Zhou C, Atlas G, Delphin E, Ye JH (2008). Labetalol facilitates GABAergic transmission to rat periaqueductal gray neurons via antagonizing β1-adrenergic receptors - a possible mechanism underlying labetalol-induced analgesia. Brain Res.

[91] Wallasch TM, Beckmann P, Kropp P (2011). Cerebrovascular reactivity during the Valsalva maneuver in migraine, tension-type headache and medication overuse headache. Funct Neurol.

[92] Dahl A, Russell D, Nyberg-Hansen R, Rootwelt K (1990). Cluster headache: Transcranial Doppler ultrasound and rCBF studies. Cephalalgia.

[93] Olesen J (2008). The role of nitric oxide (NO) in migraine, tension-type headache and cluster headache. Pharmacol Ther.

[94] Higashi Y, Sasaki S, Kurisu S, Yoshimizu A, Sasaki N, Matsuura H, Kajiyama G, Oshima T (1999). Regular aerobic exercise augments endothelium-dependent vascular relaxation in normotensive as well as hypertensive subjects: role of endothelium-derived nitric oxide. Circulation.

[95] Varin R, Mulder P, Richard V, Tamion F, Devaux C, Henry JP, Lallemand F, Lerebours G, Thuillez C (1999). Exercise improves flow-mediated vasodilatation of skeletal muscle arteries in rats with chronic heart failure. Role of nitric oxide, prostanoids, and oxidant stress. Circulation.

[96] Bigal ME, Lipton RB (2009). The epidemiology, burden, and comorbidities of migraine. Neurol Clin.

[97] French DJ, Holroyd KA, Pinell C, Malinoski PT, O’Donnell F, Hill KR (2000). Perceived self-efficacy and headache-related disability. Headache.

[98] Bromberg J, Wood ME, Black RA, Surette DA, Zacharoff KL, Chiauzzi EJ (2012). A randomized trial of a web-based intervention to improve migraine self-management and coping. Headache.

[99] Smitherman TA, Burch R, Sheikh H, Loder E (2013). The prevalence, impact, and treatment of migraine and severe headaches in the United States: a review of statistics from national surveillance studies. Headache.

[100] Steiner TJ, Birbeck GL, Jensen R, Katsarava Z, Martelletti P, Stovner LJ (2011). The global campaign, World Health Organization and lifting the burden: collaboration in action. J Headache Pain.

[101] Bloudek LM, Stokes M, Buse DC, Wilcox TK, Lipton RB, Goadsby PJ, Varon SF, Blumenfeld AM, Katsarava Z, Pascual J, Lanteri-Minet M, Cortelli P, Martelletti P (2012). Cost of healthcare for patients with migraine in five European countries: results from the international burden of migraine study (IBMS). J Headache Pain.

[102] Mitsikostas DD, Thomas AM (1999). Comorbidity of headache and depressive disorders. Cephalalgia.

[103] Deligianni CI, Vikelis M, Mitsikostas DD (2012). Depression in headaches: Chronification. Curr Opin Neurol.

[104] Verrotti A, Di Fonzo A, Penta L, Agostinelli S, Parisi P (2014). Obesity and headache/migraine: the importance of weight reduction through lifestyle modifications. Biomed Res Int.

[105] Martelletti P, Katsarava Z, Lampl C, Magis D, Bendtsen L, Negro A, Russell MB, Mitsikostas DD, Jensen RH (2014). Refractory chronic migraine: a consensus statement on clinical definition from the European headache federation. J Headache Pain.

[106] Verrotti A, Agostinelli S, D’Egidio C, Di Fonzo A, Carotenuto M, Parisi P, Esposito M, Tozzi E, Belcastro V, Mohn A, Battistella PA (2013). Impact of a weight loss program on migraine in obese adolescents. Eur J Neurol.

[107] Mauskop A (2012). Nonmedication, alternative, and complementary treatments for migraine. Continuum (Minneap Minn).

[108] Stronks DL, Tulen JHM, Bussmann JBJ, Mulder LJMM, Passchier J (2004). Interictal daily functioning in migraine. Cephalalgia.

[109] Hindiyeh NA, Krusz JC, Cowan RP (2013). Does exercise make migraines worse and tension type headaches better?. Curr Pain Headache Rep.

